# Soybean Endo-1,3-Beta-Glucanase (*GmGLU*) Interaction With *Soybean mosaic virus*-Encoded P3 Protein May Contribute to the Intercelluar Movement

**DOI:** 10.3389/fgene.2020.536771

**Published:** 2020-09-15

**Authors:** Feifei Shi, Ying Wang, Fang Zhang, Xingxing Yuan, Huatao Chen, Xuehao Chen, Xin Chen, Xiaoyan Cui

**Affiliations:** ^1^Institute of Industrial Crops, Jiangsu Academy of Agricultural Sciences/Jiangsu Key Laboratory for Horticultural Crop Genetic Improvement, Nanjing, China; ^2^Department of Plant Pathology, College of Plant Protection, China Agricultural University, Beijing, China; ^3^Department of Horticulture, College of Horticulture and Plant Protection, Yangzhou University, Yangzhou, China; ^4^Central Laboratory, Jiangsu Academy of Agricultural Sciences, Nanjing, China; ^5^Institute of Life Science, Jiangsu University, Zhenjiang, China

**Keywords:** *Soybean mosaic virus*, P3, GmGLU, interaction, callose

## Abstract

*Soybean mosaic virus* (SMV), a member of the genus *Potyvirus*, is a prevalent and devastating viral pathogen in soybean-growing regions worldwide. Potyvirus-encoded P3 protein is reported to participate in virus replication, movements, and pathogenesis. This study provides evidence that the soybean (*Glycine max*) endo-1,3-beta-glucanase protein (designated as GmGLU) interacts with SMV-P3 by using a yeast two-hybrid system to screen a soybean cDNA library. A bimolecular fluorescence complementation assay further confirmed the interaction, which occurred on the cytomembrane in *Nicotiana benthamiana* cells. Subcellular localization experiment indicated that GmGLU localized in cytomembrane and could co-localized at PD with PD marker. The transient expression of GmGLU promoted the coupling of Turnip mosaic virus replication and cell-to-cell movement in *N. benthamiana*. Meanwhile, qRT-PCR experiment demonstrated that the expression of GmGLU which involved in callose regulation increased under SMV infection. Under SMV infection, callose deposition at PD was observed obviously by staining with aniline blue, which raise a physical barrier restricting cell-to-cell movement of SMV. When overexpression the GmGLU into the leaves under SMV infection, the callose induced by SMV was degraded. Coexpression the GmGLU and SMV in soybean leaves, callose was not found, whereas a large amount of callose deposition on soybean leaves which were only under SMV infection. The results show that GmGLU can degrade the callose induced by SMV infection and indicate that GmGLU may be an essential host factor involvement in potyvirus infection.

## Highlights

- GmGLU interacted with SMV-P3 in a screening of soybean cDNAs using mY2HS.
- The interaction between GmGLU and SMV-P3 was confirmed using BiFC.
- The subcellular location of GmGLU was on the cytomembrane and form the dots at plasmodesmata sites.
- GmGLU was up-expressed in response to SMV infection.
- GmGLU overexpression of soybean decreases callose deposition.
- GmGLU overexpression promotes viral cell-to-cell movement and replication.

## Introduction

*Soybean mosaic virus* (SMV), a member of the family *Potyviridae*, is the causative agent of one of the most well-known viral diseases affecting soybean (Kim et al., 2016). Specifically, the virions are transmitted by seeds and aphids, reducing the yield and quality of soybeans (Shukla et al., 1994; Yang and Gai, 2011). The SMV genome is a single positive-stranded RNA, about 9600 nucleotides long, containing an open reading frame (ORF) and 11 different multifunctional mature proteins (Urcuqui-Inchima et al., 2001; Chung et al., 2008; Wen and Hajimorad, 2010).

P3, a 43 kDa non-structural protein, is the most variable potyviral proteins (Eiamtanasate et al., 2007). Many previous studies have shown that P3 participates in virus replication, movement, and pathogenesis (Johansen et al., 2001; Jenner et al., 2003; Suehiro et al., 2004). Some studies further revealed that the P3 cistron may also function as a virulence determinant for the SMV elicitors of Rsv1-mediated resistance (Hajimorad et al., 2006, 2008, 2011; Chowda-Reddy et al., 2010; Wen et al., 2011, 2012; Wang et al., 2014). Previous studies have revealed that the *Tobacco etch virus* P3 protein is localized in the ER membrane and forms punctate inclusions in association with the Golgi apparatus (Cui et al., 2010). Moreover, the P3 punctate structure was found to traffic along the actin filaments and co-localize with the 6 kDa peptide-containing replication vesicles (Cui et al., 2010). The potyviral P3 cistron encodes two viral proteins, P3N-PIPO and P3, which have two distinct functions: P3N-PIPO is a dedicated MP (Wei et al., 2010) and P3 is an essential component of the viral replication complexes (Cui et al., 2017). Furthermore, P3N-PIPO interacts with P3 via the shared N-terminal domain to recruit viral replication vesicles for cell-to-cell movement (Chai et al., 2020). Therefore, we speculate that P3 may act as a bridge between virus replication and movement.

Previous studies have shown that P3 targets the host elongation factor eEF1A, inducing the associated unfolded protein response, which in turn facilitates SMV replication (Luan et al., 2016). It has been reported that P3 interacts with the small subunit of ribulose-1,5-bisphosphate carboxylase/oxygenase (RubisCO), thus contributing to the development of viral infection symptoms in host plants (Lin et al., 2011). Furthermore, previous reports have studied the interaction of soybean proteins with P3, including soybean actin-depolymerizing factor 2 (Lu et al., 2015), hypersensitive response-like lesion-inducing protein (Luan et al., 2019), endoplasmic reticulum homologous protein (GmRHP) (Cui et al., 2018), which might play important role in SMV infection. Taken together, these studies suggest that studying the function of P3 and its interactors can help us better understand viral infection.

Endo-1,3-β-glucanases (GLU) are grouped in the PR-2 family of PR proteins (Balasubramanian et al., 2012; Piršelová and Matušíková, 2013), which catalyze the hydrolysis of 1,3-β-glucan linkages and play important roles in plant defense and development. For instance, the previously reported that abscisic acid inhibits the transcription of 1,3-β-glucanase and thus remarkably strengthens the ability of plants to resist viruses (Mauch-Mani and Mauch, 2005; Fernández-Crespo et al., 2017). Callose, a 1,3-β-glucan which is a common substrate of GLU, is reversibly and transiently deposited in plant cell walls as part of the hypersensitive response to fungi, viruses, and abiotic stresses (Stone and Clarke, 1992; Kauss, 1996). Callose deposition occurs in the wall surrounding plasmodesmata [the intercellular connections between plant cells that allow cell-to-cell transport of sugars, amino acids, inorganic ions, proteins, and nucleic acids (reviewed by Lucas et al., 1993)] at both ends of the channel, compressing the plasma membrane inward, thus creating a narrowed neck region (Radford et al., 1998), which inhibits the transport of macromolecules through plasmodesma (PD). GLU as hydrolytic enzymes which specifically degrade callose, change the dynamic balance between callose synthesis and degradation, while promoting cell-to-cell viral movement and spread. Thus, callose is thought to act as a physical barrier limiting or preventing the spread of viruses in the host (Allison and Shalla, 1974; Iglesias and Meins, 2000). Previous study has shown that increased expression of GLU I in virus-infected cells can increase the size of local lesions and in the absence of the hypersensitive response, can promote virus spread (Gregor et al., 2001). As opposed to GLU overexpression, the deficiency in GLU results in reduced susceptibility to viral infection due to net increase in PD callose levels (Zavaliev et al., 2011).

In this study, we demonstrated that the soybean factor endo-1,3-β-glucanase (designated as GmGLU) interacts with SMV P3 in a mY2H analysis and BiFC. *GmGLU* transformed and transiently expressed into *Nicotiana benthamiana* leaf epidermal cells by agroinfiltration, formed punctate inclusions on the cytomembrane, and co-located with a PD marker at the PD in *N. benthamiana*. The expression of GmGLU in soybean leaves inoculated with SMV was higher than that in leaves mock-inoculated with PBS at 48 and 72 h post-infiltration. Furthermore, we demonstrated that overexpression of soybean *GLU* can decrease callose deposition at the PD which was induced by SMV. We also observed that the viral spread speed and genomic RNA accumulation significantly increased when coexpression GmGLU in *N. benthamiana* leaves. Our data support that GmGLU plays an important role in viral infection.

## Materials and Methods

### Plants, Viral Strains, and Plasmids

Seeds of *Glycine max* ‘Nannong 1138-2’ were obtained from the Soybean Improvement Center of Nanjing Agricultural University (Nanjing, China). Leaves of 2-weeks-old ‘Nannong 1138-2’ plants were inoculated via a point inoculation approach or direct rubbing by a brush or toothpick with SMV, isolate 6067-1 (strain SC15) (GenBank Accession: JF833015.1) in controlled environment chambers at 22°C with a 16-h light/8-h dark photoperiod. The *N. benthamiana* seedlings were grown in growth chambers under a 16-h light/8-h dark photoperiod at 22 to 24°C. *Escherichia coli* strain DH5a, *Saccharomyces cerevisiae* strain NMY51 (Dualsystems Biotech, Zurich, Switzerland), yeast expression vectors (pPR3-N, and pBT3-STE) (Dualsystems Biotech), and the pPR3-gateway vector (modified from pPR3-N) were used in this study. All vectors were constructed using Gateway technology. *Agrobacterium tumefaciens* strain EHA105 and the fluorescence expression vectors pEarleyGate104, pEarleyGate201-YC, pEarleyGate202-YN and the plant expression vector pEarleyGate201 were stored at the Jiangsu Province Academy of Agricultural Sciences (Nanjing, China).

### Gene Cloning and Plasmid Construction

Gateway technology (Invitrogen, Burlington, ON, Canada) was used to generate all plasmid clones used in this work. Gene sequences were amplified by PCR using PrimeSTAR HS DNA Polymerase (Takara, Dalian, China). The resulting DNA fragments were purified and transferred by recombination into the entry vector pDONR221 (Invitrogen) using BP clonase II (Invitrogen) following the supplier’s recommendations. Insertions in the resulting pDONR clones were verified by DNA sequencing. The destination vectors, yeast expression vector (pPR3-gateway) or fluorescence expression vectors (pEarleyGate104, pEarleyGate201-YC, and pEarleyGate202-YN), or plant expression vector (pEarleyGate201) were produced using LR clonase II (Invitrogen) according to the conditions and procedures recommended by the supplier. The primer sequences used in this study are listed in Table 1.

**TABLE 1:** Primer sequences used for cloning GLU genes.

Name	Sequence (5′→ 3′)^a^
SMV-P3-F	ATTAACAAGGCCATTACGGCCGGGGAAGTGCAACAAAGGATGA
SMV-P3-R	AACTGATTGGCCGAGGCGGCCCCCTGTGCGGAGACATCTTCT GA
GmGLU-F	GGGGACAAGTTTGTACAAAAAAGCAGGCTTCATGATTTTCTCAA GAGGCAAC
GmGLU-R	GGGGACCACTTTGTACAAGAAAGCTGGGTCATTGAAACTGAG TTGGTATTTAGGT

*^a^The restriction enzyme sequences or gateway sequences are underlined.*

### mY2H Analysis

The mY2H tests were performed using the Matchmaker DUAL membrane system (Clontech, Palo Alto, CA, United States) according to the manufacturer’s protocol. The DUAL membrane system was developed to identify and characterize protein–protein interactions between integral membrane proteins, membrane-associated proteins, and soluble proteins in their natural condition. The fusion vector pPR3-gateway-GmGLU was constructed by inserting the GmGLU gene into the pPR3-gateway vector. pPR3-gateway-GmGLU and pBT3-STE-P3 (constructed previously) were co-transformed into yeast cells using the small-scale lithium acetate transformation method. The yeast cells were then grown on SD/-Leu/-Trp and SD/-Ade/-His/-Leu/-Trp medium for 3–7 days at 30°C. Colonies growing on SD/-Ade/-His/-Leu/-Trp medium were transferred to SD/-Ade/-His/-Leu/-Trp/X-α-Gal solid medium and grown for 3–5 days at 30°C. Blue colonies were considered as positive clones. Yeast cells co-transformed with empty pBT3-STE-P3+pPR3-gateway, pBT3-STE+pPR3-gateway-GmGLU, and pBT3-STE+pPR3-gateway were used as a negative control, and those co-transformed with pBT3-STE-P3N-PIPO+pPR3-gateway-GmGOS1 were used as a positive control (Song et al., 2016). The experiments were repeated at least three times.

### Agroinfiltration in *N. benthamiana*

All experiments were performed using *N. benthamiana*, an experimental host of SMV. For the BiFC assay, the *A. tumefaciens* strain EHA105 was transformed with pEarleyGate202-YN-GmGLU and pEarleyGate201-YC-P3 via cryoapplication. For the subcellular localization assay or overexpression, *A. tumefaciens* strain EHA105 was transformed with pEarleyGate104-GmGLU or pEarleyGate201-GmGLU, then cultured overnight with appropriate antibiotics at 28°C (Sparkes et al., 2006). For intercellular movement analysis, the infectious clone TuMV-GFP was described in Cui et al. (2017). The *Agrobacterium* cells were diluted to an optical density (OD_600_) of 0.5–0.6 for BiFC experiments (Tian et al., 2011) and 0.1–0.2 for subcellular expression and 0.001 for intercellular movement analyses. The diluted *A. tumefaciens* was injected into *N. benthamiana* and fluorescence was observed by confocal microscopy. Each BiFC experiment, subcellular localization assay and intercellular movement analysis was repeated at least three times, and each treatment included at least three independent *N. benthamiana* plants.

### RNA Extraction and RT-PCR

RNA was isolated from 50 to 100 mg of frozen soybean leaf tissue using the total RNA isolation kit according to the manufacturer’s instructions (TIANGEN, Nanjing, China). The quality and quantity of RNA were measured with a nucleic acid and protein detection instrument. Reverse transcription (RT) and first-strand cDNA synthesis were carried out using Fast-RT SuperMix (TIANGEN, Nanjing, China). The primer pair SMV-CP-F (5′-ATGGTTGAGAGGGAAAGATCTATCTC-3′) and SMV-CP-R (5′-AGAGCTGCAGCCTTCATCTTG-3′) was used to detect soybean genomic RNA. An SMV coat protein gene DNA fragment at the expected size of 500 bp was amplified as follows: initial denaturation at 94°C for 2 min followed by 30 cycles of denaturation at 94°C for 0.5 min, annealing at 55°C for 0.5 min and elongation at 72°C for 1 min. The final extension step was at 72°C for 10 min.

### Quantitative Reverse Transcription PCR

Quantitative reverse transcription PCR was carried out in a LightCycler 480 (Roche, Penzberg, Germany) using SYBR^®^ Green I (Takara, Dalian, China). The primer pair GmGLU-F (5′-TATTTAGGTGATTTGTCAGGCC-3′) and GmGLU-R (5′-CCGCAATTTGATTAATCATGCC-3′) was used to detect soybean genomic RNA, and the primer pair GmTubulin-F (5′-CGAGTTCACAGAGGCAGAG-3′) and GmTubulin-R (5′-CACTTACGCATCACATAGCA-3′) was used to detect the reference gene Tubulin in *G. max*, which was used for normalization. The primer pair TuCP-F (5′-GGCACTCAAGAAAGGCAAGG-3′) and TuCP-R (5′-CTCCGTCAGTTCGTAATCAGC-3′) was used for detection of TuMV viral genomic RNA, and the primers NbActin-F (5′-GGGATGTGAAGGAGAAGTTGGC-3′) and NbActin-R (5′-ATCAGCAATGCCCGGGAACA-3′) for the reference gene *Actin* in *N. benthamiana* were used for normalization. The final reaction mixtures contained 250 nM of each primer in a volume of 20 μL. All genes were amplified under the same reaction conditions: 30 s at 95°C, followed by 40 cycles of 10 s at 95°C, 30 s at 53°C, and 34 s at 72°C. The specificity of amplification was verified using a melting curve analysis (60–95°C). qRT-PCRs with no template control were performed under the same conditions. All reactions were performed in triplicate, and the mean value was calculated. Cycle threshold (CT) values were determined using LightCycler 480 Real-Time PCR software (Roche, Penzberg, Germany) and were used for gene expression analysis. Bar charts and error lines were created using OriginLab OriginPro 8.5.

### Aniline Blue Staining

Callose was stained by a modification of the aniline blue fluorochrome method (Conrath et al., 1998; Li et al., 2012). In brief, sections of about 1 cm × 1 cm were cut at different time points after inoculation from unifoliate leaves, which do not have large veins. Chlorophyll was cleared by heating the samples at 55°C in lactophenol (water: glycerol: phenol: lactic acid, 1:2:2:1 by volume) diluted with two volumes of ethanol for 30 min. The cleared leaf samples were stained in the dark with 0.1% aniline blue (Macklin, China) in 0.15M K_2_HPO_4_ buffer, pH 9.0, for 30–60 min. The leaves were observed under a confocal microscope. The experiment was repeated at least three times, and each treatment included at least three independent ‘Nannong 1138-2’ plants.

### Confocal Microscopy

Epidermal cells of *N. benthamiana* were examined with Ultra View VOX inverted confocal microscope. For confocal microscopy, CFP was excited at 458 nm, and the emitted light was captured at 440 to 470 nm; YFP was excited at 514 nm, and the emitted light was captured at 525 to 650 nm; GFP was excited at 488 nm and the emitted light was captured at 505 to 555 nm. Post-acquisition images were processed by Adobe Photoshop software.

## Results

### Interaction Between GmGLU and SMV-P3 in Y2HS

**FIGURE 1:**
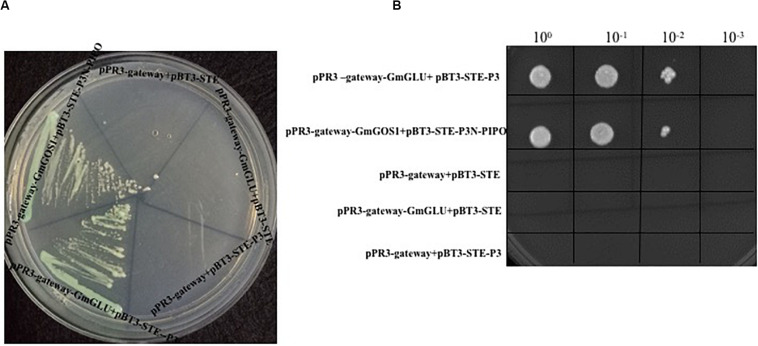
Yeast two-hybrid assay to determine interaction between SMV P3 and GmGLU in *Saccharomyces cerevisiae* NMY51 cells using plasmids pPR3-gateway-GmGOS1 and pBT3-STE-P3N-PIPO (as positive control), pBT3-STE-P3+pPR3-gateway, pBT3-STE+pPR3-gateway-GmGLU, pPR3-gateway+pBT3-STE (as negative control), and pBT3-STE-P3+pPR3-gateway-GmGLU for transformation of yeast cells followed on SD/-Ade/-His/-Leu/-Trp medium containing X-α-Gal **(A)**. Dilutions of pPR3-gateway-GmGLU+pBT3-STE-P3, positive control and negative control on SD/-Ade/-His/-Leu/-Trp medium **(B)**.

In a mY2HS analysis using the SMV-P3 protein as bait and a soybean cDNA library as prey, one candidate gene was identified and shared a 94.65% nucleotide sequence identity with the *G. max* endo-1,3-β-glucanase (AY461847.1). Thus, this isolated soybean factor was named GmGLU. The gene encoding SMV-P3 was inserted into pBT3-STE and the complete cDNA of soybean GmGLU (Accession Number: AY461847.1) was inserted into pPR3-gateway. The clones harboring pPR3-gateway-GmGLU+pBT3-STE-P3 grew well on SD/-Ade/-His/-Leu/-Trp/X-α-Gal medium (Figure 1A). Negative control P3N-PIPO cannot interact with GmGLU (Supplementary Figure S1). The positive control harboring pPR3-gateway-GmGOS1+pBT3-STE-P3N-PIPO also grew well on the selective medium, while the negative controls harboring pPR3-gateway-GmGLU+pBT3-STE\pPR3-gateway+pBT3-STE-P3 and pPR3-gateway+pBT3-STE did not grow on the selective medium. Different dilutions of the cells harboring pPR3-gateway-GmGLU+pBT3-STE-P3 grew as well as the positive control on SD/-Ade/-His/-Leu/-Trp medium (Figure 1B).

### Confirmation of Interaction by BiFC

**FIGURE 2:**
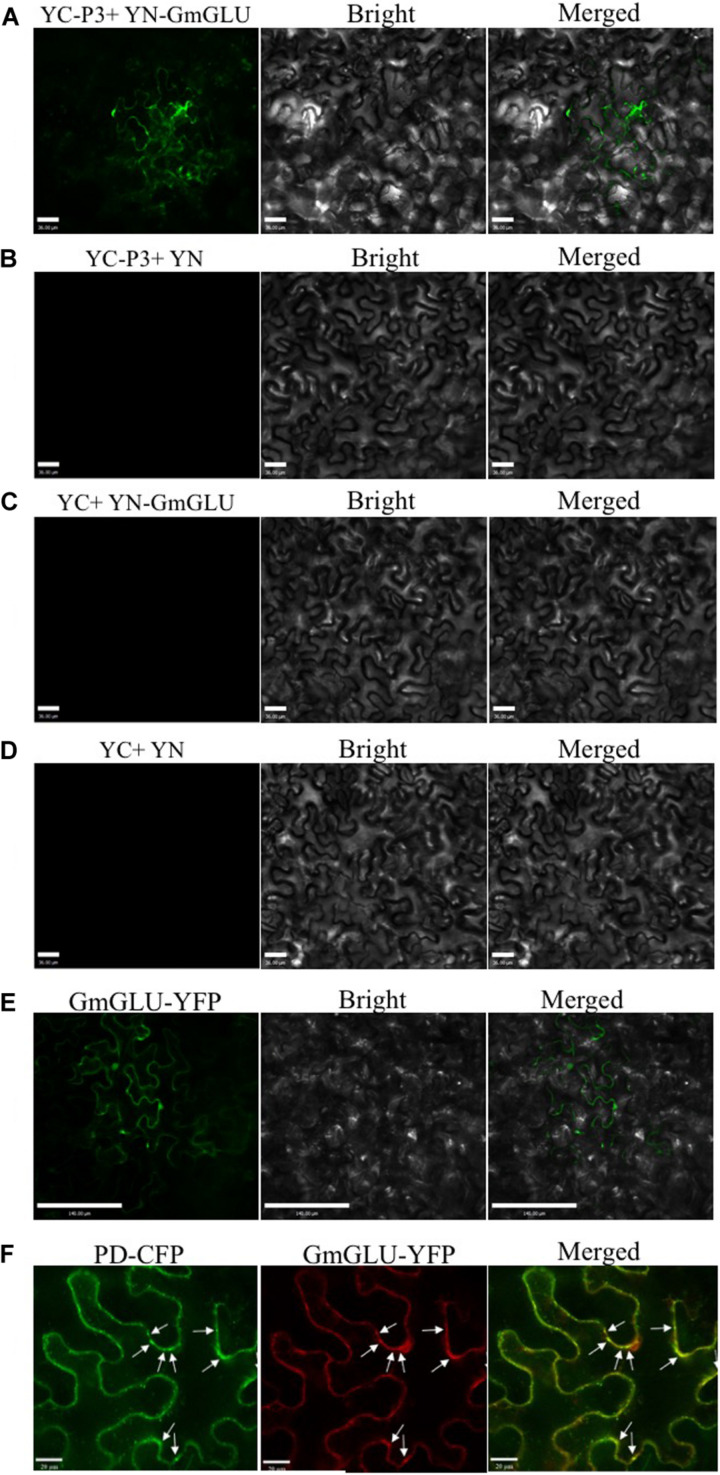
Bimolecular fluorescence complementation assays demonstrating SMV P3 and GmGLU interaction in cytomembrane and epidermal cells of *N. benthamiana*. **(A)** SMV P3 and GmGLU were fused to N- and C-terminal of yellow fluorescent protein (YFP) respectively. YFP fluorescence in *N. benthamiana* leaves agroinfiltrated with pEarleyGate202-YN-GmGLU+pEarleyGate201-YC-P3. **(B–D)** Negative controls in the BiFC assay of GmGLU and SMV P3 interaction. Bar indicates 36 μm. **(E)** Fluorescence of YFP fused to GmGLU protein of host in *N. benthamiana* leaves under spectral confocal laser microscope. Bar indicates 140 μm. **(F)** Co-expression of plasmodesmata (PD) marker fused with cyan fluorescent protein (CFP) and GmGLU protein fused with YFP. GmGLU protein accumulated in dots on cytomembrane (PD marker sites). Bar indicates 20 μm.

To further confirm the interaction between SMV-P3 and GmGLU *in vivo*, we recombined GmGLU into pEarlygate202-YN to create a fusion construct containing the N-terminal fragment of yellow fluorescent protein (YFP) and recombined SMV-P3 into pEarlygate201-YC to create a fusion construct containing the C-terminal fragment of YFP. These two vectors were co-transformed into *N. benthamiana* via *Agrobacterium*-mediated transformation, allowing the transient expression of fusion proteins. The green fluorescence indicative of the P3 and GmGLU combination was observed on the cytomembrane at 48 hpi (Figure 2A). No BiFC fluorescence was detected in the negative control samples expressing combinations with non-hybrid YC or/and YN (Figures 2B–D). These results confirmed that GmGLU could interact with SMV-P3 *in vivo*.

### Subcellular Localization of GmGLU in *N. benthamiana* Cells

To explore the possible functions of GmGLU, we analyzed its subcellular localization in *N. benthamiana* leaf epidermal cells. The gene was fused with YFP to form pEarleyGate104-GmGLU and agroinfiltrated into *N. benthamiana*. Using fluorescence microscopy at 48 hpi, the GmGLU protein fluoresced on the cytomembrane and formed small dots (Figure 2E). To investigate that where is small dots, the PD marker fused with cyan fluorescent protein (Wei et al., 2010; Park et al., 2017) and GmGLU protein fused with YFP coexpress in *N. benthamiana* leaf epidermal cells. The punctate inclusions on the cytomembrane from GmGLU were observed to overlap with PD marker on the points (Figure 2F). These results indicated that GmGLU was located at the PD site in leaf cells of *N. benthamiana*.

### SMV Infection Induced the Overexpression of GmGLU

**FIGURE 3:**
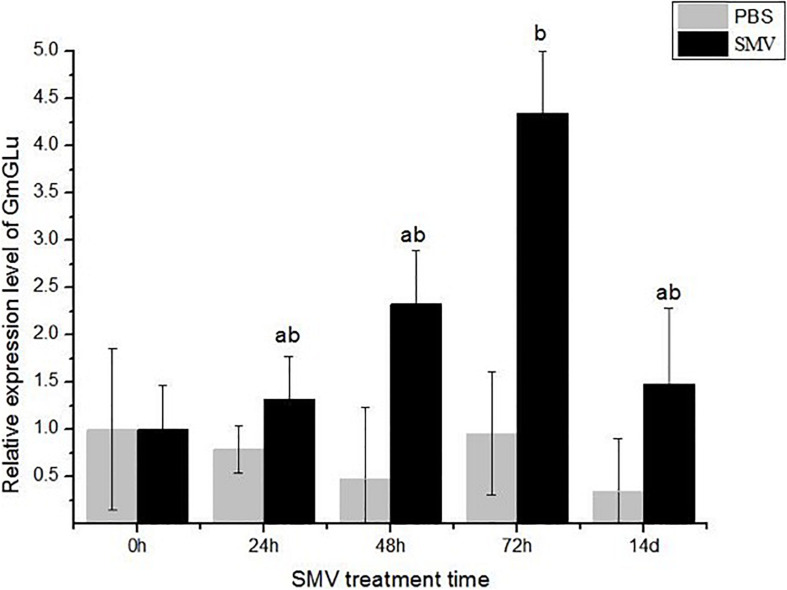
qRT-PCR analysis of GmGLU expression levels. Y-axes show relative expression levels of genes in SMV-infected and mock (PBS)-infected samples at different time points. Each error bar represents mean ± SD. Alphabetic symbols represent statistical significance: ^ab^0.01 < P ≤ 0.05; ^b^*P* ≤ 0.01.

To investigate whether GmGLU is involved in response to SMV infection, we inoculated SMV onto the soybean variety ‘Nannong 1138-2’ and then detected the expression level of GmGLU at time-course after infection. The expression level of GmGLU in soybean leaves inoculated with SMV were significantly higher than those in leaves mock-inoculated with PBS at 24, 48, 72 hpi and 14 dpi (Figure 3). Additionally, a significant difference (*P* < 0.01) was found between the leaves treatment by SMV and PBS at 72 hpi (Figure 3). These findings confirmed that the infection of SMV induced the overexpression of GmGLU in soybean leaves.

### SMV Infection Induces Callose Deposition

**FIGURE 4:**
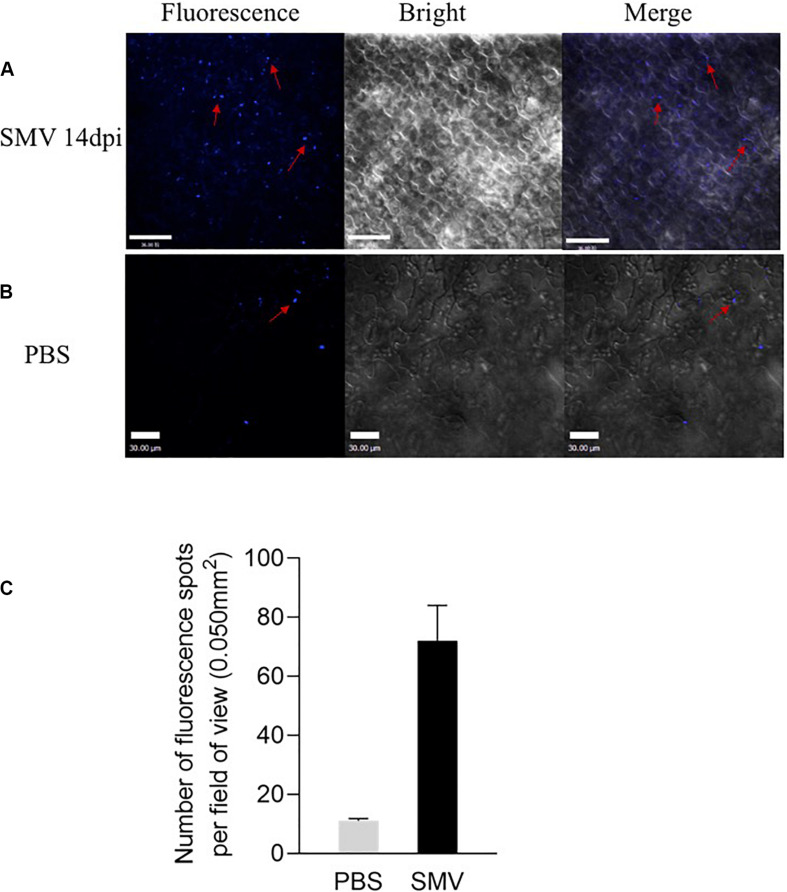
Fluorescence spots at the inoculation sites detected from soybean cv. Nannong 1138-2 leaves inoculated with SMV strain SC-15. **(A)** Lots of Fluorescence spots were detected as uneven distribution on the cell membrane 14 days post-inoculation. **(B)** A few of callose deposition, caused by friction was observed as negative control. Bar indicates 30 μm. **(C)** Number of fluorescence spots per field of view (0.050 mm^2^), as indicated callose deposition in systemically infected leaves at 14 dpi. Three plants were examined in each treatment, two small areas of each plant were inoculated, and five fields of view are randomly observed in each area. Error bars represent the standard deviation from 30 fields of view and data were from three independent experiments.

Callose can modulate the PD permeability, and the deposition of callose is one of the critical defense responses of host plants against viral infection. In order to detect the effect of SMV infection on callose deposition, we inoculated SMV into the soybean variety ‘Nannong 1138-2’ and then detected the deposition of callose 14 days after infection. PBS treatment was used as a negative control. Many points with blue fluorescence, unevenly distributed on the cell membrane, were observed by confocal microscopy (Figures 4A,C). The control group had a few points of callose deposition, caused by friction (Figures 4B,C). The accumulation of callose was quantified with the software ImageJ and was shown in Figure 4C. These results suggested that SMV infection can significantly induce callose deposition.

### GmGLU Decreases Callose Deposition at the PD

**FIGURE 5:**
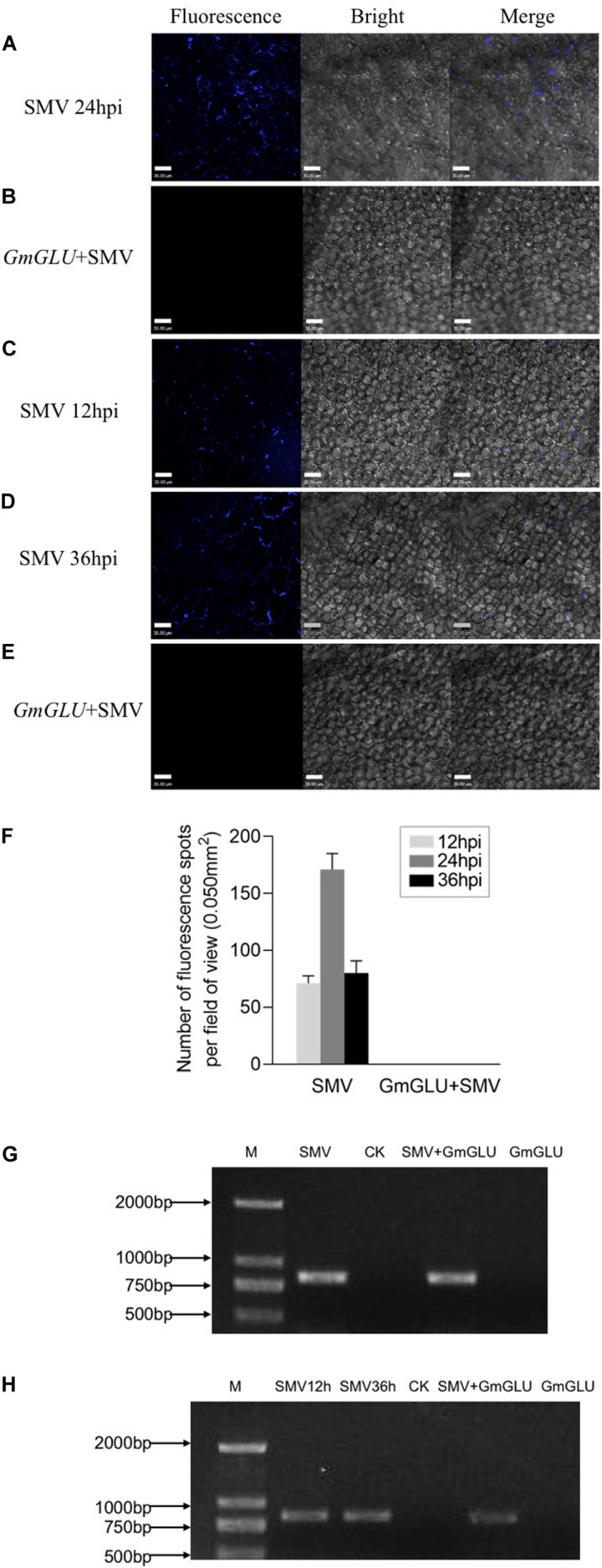
GmGLU decreases callose deposition induced by SMV infection. GmGLU in the soybean leaves was overexpressed prior to the inoculation with SMV. **(A)** Confocal microscopic observation. There was a large amount of callose deposition on soybean leaves which were only infected by SMV. **(B)** There was no callose deposition on soybean leaves with overexpression GmGLU. We observed callose deposition through overexpressing GmGLU into soybean leaves which inoculated by SMV. **(C,D)** There were many the fluorescence spots formed by callose at 12 h and 36 post-SMV inoculation. **(E)** The fluorescence formed by callose at the inoculation point were disappeared following the transfection of GmGLU. Bar indicates 30 μm. **(F)** Number of fluorescence spots per field of view (0.050 mm^2^), as indicated callose deposition in primarily infected leaves. Three plants were examined in each treatment, two small areas of each plant were inoculated, and five fields of view are randomly observed in each area. Error bars represent the standard deviation from 30 fields of view and data were from three independent experiments. **(G,H)** Detection of viral CP-RNA by RT-PCR analysis in the upper leaves. The viral CP-RNA was detected in the SMV-infected soybean cv. Nannong 1138-2 (G: lane 1, lane 3; H: lane 1, lane 2, lane 4). The viral CP-RNA was not detected from the negative control and GLU-treatment (G: lane 2, lane 4; H: lane 3, lane 5). Independent experiments were performed three times.

To further examine whether the soybean GmGLU plays a role in callose deposition at the PD, we inoculated SMV by toothpick on the leaves of soybean, where have been agroinfiltrated with GmGLU 24 h in advance. The leaves without agroinfiltration of GmGLU were used as control. After 24 h, we observed there was no callose deposition on soybean leaves with GmGLU overexpression (Figures 5B,F), whereas large callose depositions were found on soybean leaves which were only infected by SMV (Figures 5A,F). We therefore speculated that GmGLU inhibited the deposition of callose.

To further confirm this hypothesis, we observed callose deposition through overexpressing GmGLU into SMV-infected leaves. At 12 h post-SMV inoculation, a few fluorescence spots resulting from callose formation were found by staining with aniline blue (Figures 5C,F). We then transfected GmGLU into the SMV-infected leaves. At 36 h post-SMV inoculation, we found that the fluorescence formed by callose at the inoculation point disappeared following the transfection of GmGLU (Figures 5E,F), while there was considerable callose deposition on soybean leaves which were only infected with SMV (Figures 5D,F). No fluorescent spots produced by callose deposition was detected on leaves only inoculated with GmGLU and healthy plant leaves (Supplementary Figures S2A,B). This result suggests that GmGLU could degrade callose. Further, the viral RNA on the leaves of the inoculation was detected by RT-PCR analysis to prove that the plants were indeed infected by SMV (Figures 5G,H). These results indicated that the overexpression of soybean GmGLU decreases callose deposition induced by SMV infection at the PD.

### Overexpression of GmGLU Promotes Viral Proliferation

**FIGURE 6:**
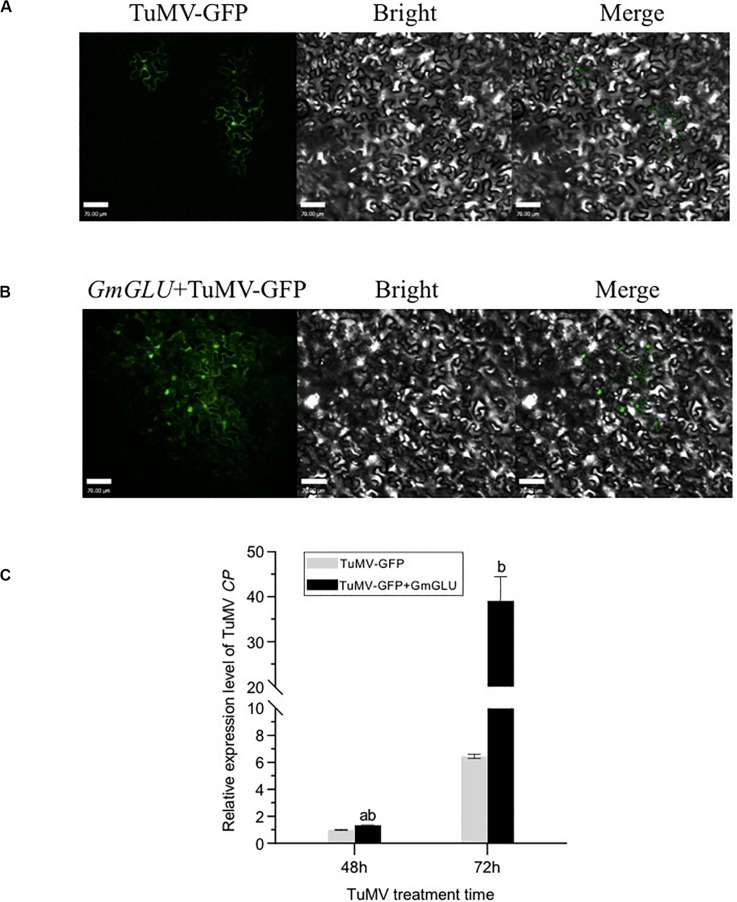
Expression of GmGLU promote the viral proliferation. **(A,B)** Confocal microscopic observation of viral intercellular movement of TuMV-GFP, TuMV-GFP/GmGLU-201. Images were taken 3.5 days post-agroinfiltration. Independent experiments were performed three times. Typical imaging was shown. Bar indicates 70 μm. **(C)** qRT-PCR detection of the viral accumulation level in *N. benthamiana* leaves transfected with TuMV-GFP, TuMV-GFP/GmGLU-201. Leaves were collected at 48 h post-transfection. Viral RNA was quantified by qRT-PCR analysis of the CP RNA level using the *N. benthmiana* ACTIN gene transcripts as the internal control. TuMV-GFP genomic RNA level was normalized to 1. Statistical differences from three biological replicates, determined by unpaired two-tailed Student’s test, are indicated by ab and b as follows: ^ab^0.001 < P ≤ 0.01; ^b^*P* ≤ 0.001. Each error bar represents mean ± SD.

Systemic viral spread was monitored with a recombinant form of TuMV-GFP (Cui et al., 2017). The infectious clone TuMV-GFP and pEarleyGate201-GmGLU were agroinfiltrated into *N. benthamiana* leaf cells, and the spread of fluorescence due to TuMV-GFP was monitored 3.5 days later. As Figure 6B shows, leaves co-infected with TuMV-GFP/GmGLU-201 showed a cluster of virus-infected cells, while sporadic luminescence occurred at the site of the initial infection in *N. benthamiana* leaves agroinfiltrated with the control TuMV-GFP (Figure 6A). The virus spread significantly increased of the GLU-treated plants compared with the control (Figure 6B). These results suggested that the movement of viruses is facilitated in the plant with GmGLU overexpression.

To test if GmGLU overexpression affects virus accumulation, we carried out qRT-PCR assay. *N. benthamiana* leaves were inoculated with the infectious clone TuMV-GFP and GmGLU-201, qRT-PCR was performed to quantify TuMV genomic RNA 48 and 72 h post-transfection. We found that GmGLU overexpression significantly promotes TuMV genomic RNA accumulation compared with control leaves which were only agroinfiltrated by TuMV-GFP (Figure 6C). Taken together, these results indicated that GmGLU overexpression promotes viral cell-to-cell movement and accumulation.

## Discussion

*Soybean mosaic virus* is composed of a single-stranded, positive-sense RNA genome and coat protein (CP) that protects nucleic acid. Since viruses lack their own reproduction system, they achieve systemic infection by interacting with host proteins by taking advantage of the host’s cellular mechanisms for replication and intracellular/intercellular movement **(Gergerich and Dolja, 2006)**. If the virus loses the ability to move, infection will be limited to an initial infection site or infected organ (Hong and Ju, 2017).

Previous reports indicated that the successful infection of plants by most viruses depends on cell-to-cell movement via PD and rapid long distance movement through the sieve tubes of the phloem (Lucas, 1995; Van Bel, 2003; Lucas and Lee, 2004; Oparka, 2004). For plant viruses, the ability of crossing cell walls through PD into adjacent healthy cells is necessary to establish systemic infection (Carrington et al., 1996; Seron and Haenni, 1996). However, virions are too large to move through the PD, and thus transport through PD is usually limited to small molecules less than 1 kDa in size (Lucas, 1995). To overcome this limitation, most viruses encode MPs, which interact with host proteins to increase the SEL of the PD and help virions pass through PD into adjacent cells (Lucas, 1995; Iglesias and Meins, 2000). Our results suggest that GLU interacts with P3 to reduce the SEL, which can play a role in potyviral intercellular spread in infected plants.

Callose is a linear 1,3-β-glucan playing an important role on the plant allergy defense response (Piršelová and Matušíková, 2013). The callose content at PD determines the aperture of the PD channel and thus controls cell-to-cell transport of macromolecules (Bucher et al., 2001; Levy and Epel, 2009; Zavaliev et al., 2011). Once the accumulated amount of callose is down-regulated, it will cause PD to relax and allow more macromolecules trafficking (Beffa et al., 1996; Iglesias and Meins, 2000). The net callose level at PD is determined by the balance between the activity of callose synthase and 1,3-β-glucanase, because loss of either enzyme activity affects callose accumulation and transport through PD (Guseman et al., 2010; Vaten et al., 2011). The accumulation of callose in susceptible hosts is very low or similar to uninfected plants (Krasavina et al., 2002; Zavaliev et al., 2011). In this study, we explored the mechanism by which GLU degrades callose by targeting P3, and our results provide evidence for the regulatory effect of GmGLU on callose under SMV infection.

In this study, GmGLU interacted with SMV-P3 in a screening of soybean cDNAs using mY2HS. The expression of *GmGLU* in soybean leaves inoculated with SMV was increased. GLU is a PR protein, located on the cytomembrane and forms the dots at plasmodesmata sites, playing important roles in plant defense and development (Leubner-Metzger and Meins, 1999; Wan et al., 2011). GLU are cell wall enzymes that catalyze the callose and enhances viral transmission by degrading callose on PD (Deom et al., 1992; Zavaliev et al., 2011). The role of the GLU in virus spread has been described in TMV (Bucher et al., 2001; Gregor et al., 2001). Infection of tobacco with TMV led to a 20-fold increase in class I GLU levels (Vogeli-Lange et al., 1988; Epel, 2009). Furthermore, by combining SMV infection with aniline blue staining, we demonstrate that callose deposition induced by SMV infection was decreased when soybean *GLU* is overexpressed. The viral spread speed and genomic RNA accumulation significantly increased under overexpression of *GmGLU*. This conclusion is consistent with the research of Bucher et al. (2001). It is widely believed that the deposition of callose is a critical defense response of host plants against viral infection (Li et al., 2012). These data suggest that GmGLU regulate the callose deposition at the PD to allow viral intercellular trafficking in viral infection by interacting with SMV-P3.

## Data Availability Statement

Publicly available datasets were analyzed in this study. This data can be found at NCBI (AY461847.1).

## Author Contributions

XYC conceived the project. XYC and XC supervised the work. FS and YW performed most of the experiments with assistance from FZ, XY, HC, and XHC. YW, FS, and XYC wrote the manuscript with contributions from all the authors. All authors analyzed the data.

## Conflict of Interest

The authors declare that the research was conducted in the absence of any commercial or financial relationships that could be construed as a potential conflict of interest.

## Supplementary Material

FIGURE S1 Yeast two-hybrid assay used to determine that SMV P3N-PIPO and GmGLU cannot interact in *Saccharomyces cerevisiae* NMY51 cells using plasmids pPR3-gateway-GmGLU+pBT3-STE-P3 (1) and pPR3-gateway-GmGOS1+pBT3-STE-P3N-PIPO (2) as positive control, pPR3-gateway+pBT3-STE-P3 (4) and pPR3-gateway+pBT3-STE-P3N-PIPO (5) and pPR3-gateway-GmGLU+pBT3-STE (6) and pPR3-gateway+pBT3-STE (7) as negative control, pPR3-gateway-GmGLU+pBT3-STE-P3N-PIPO (3) for transformation of yeast cells followed on SD/-Ade/-His/-Leu/-Trp medium containing X-α-Gal. In addition, negative controls 4–7 and pPR3-gateway-GmGLU+pBT3-STE-P3N-PIPO could not grow on the selective medium.

FIGURE S2 No fluorescent spots produced by callose deposition were detected on leaves only inoculated with GmGLU and healthy plant leaves. **(A)** No callose deposits can be detected on leaves overexpressing GmGLU. **(B)** No callose deposits can be detected on healthy plant leaves. Bar indicates 30 μm.

## Abbreviations

BiFC: bimolecular fluorescence complementation
ER: endoplasmic reticulum
GmGLU: soybean (*Glycine max*) endo-1,3-beta-glucanase
MP: movement protein
mY2HS: membrane yeast two-hybrid system
PBS: phosphatic buffer solution
PD: plasmodesmata
PR: pathogenesis-related
PVX: *Potato virus X*
PVY: *Potato virus Y*
SEL: size exclusion limit
SMV: *Soybean mosaic virus*
TMV: *Tobacco mosaic virus*
TuMV: *Turnip mosaic virus*.
