# Supplementary figures and images for: Soybean Endo-1,3-Beta-Glucanase (GmGLU) Interaction With Soybean mosaic virus-Encoded P3 Protein May Contribute to the Intercelluar Movement
Source: Front Genet. 2020 Sep 15;11:536771. doi: 10.3389/fgene.2020.536771 (PMC7522550; doi:10.3389/fgene.2020.536771)

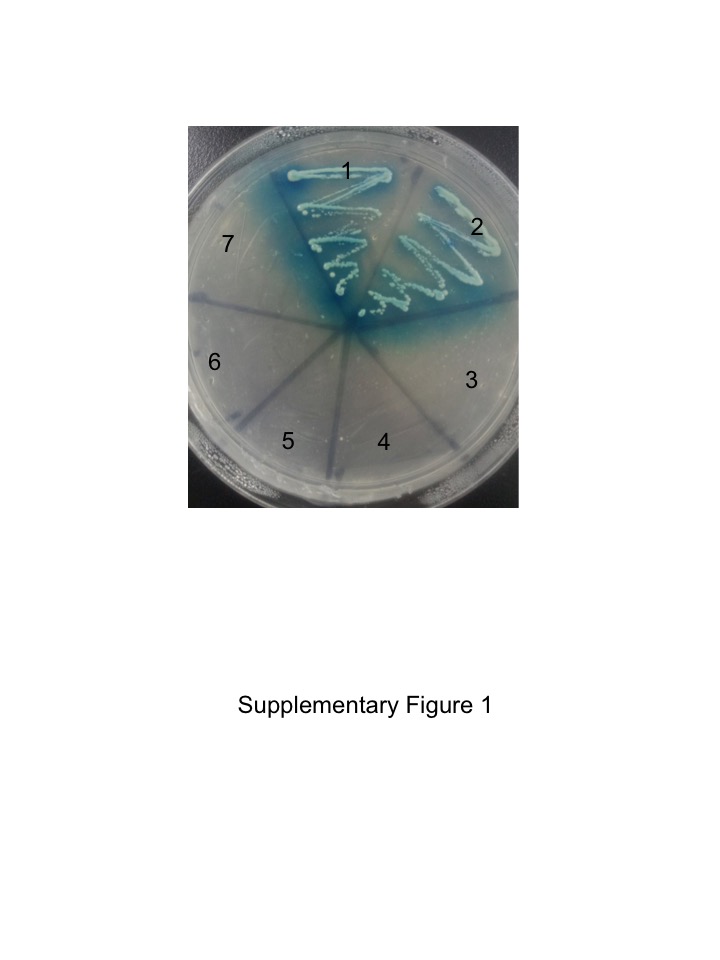

Supplement: FIGURE S1 — Yeast two-hybrid assay used to determine that SMV P3N-PIPO and GmGLU cannot interact in Saccharomyces cerevisiae NMY51 cells using plasmids pPR3-gateway-GmGLU+pBT3-STE-P3 (1) and pPR3-gateway-GmGOS1+pBT3-STE-P3N-PIPO (2) as positive control, pPR3-gateway+pBT3-STE-P3 (4) and pPR3-gateway+pBT3-STE-P3N-PIPO (5) and pPR3-gateway-GmGLU+pBT3-STE (6) and pPR3-gateway+pBT3-STE (7) as negative control, pPR3-gateway-GmGLU+pBT3-STE-P3N-PIPO (3) for transformation of yeast cells followed on SD/-Ade/-His/-Leu/-Trp medium containing X-α-Gal. In addition, negative controls 4–7 and pPR3-gateway-GmGLU+pBT3-STE-P3N-PIPO could not grow on the selective medium. [file Image_1.JPEG]

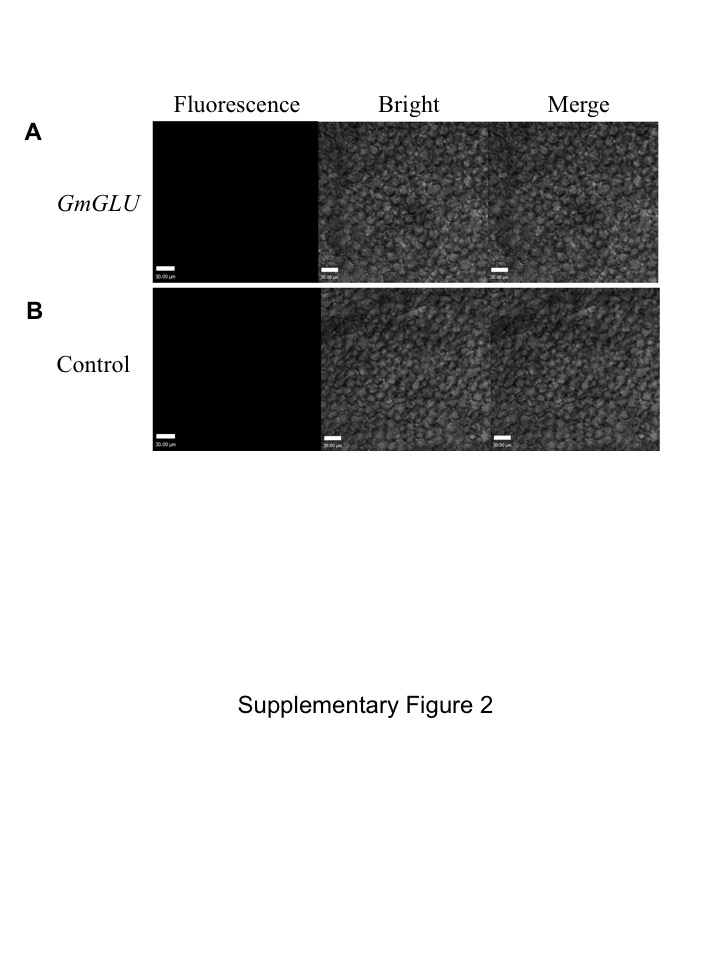

Supplement: FIGURE S2 — No fluorescent spots produced by callose deposition were detected on leaves only inoculated with GmGLU and healthy plant leaves. (A) No callose deposits can be detected on leaves overexpressing GmGLU. (B) No callose deposits can be detected on healthy plant leaves. Bar indicates 30 μm. [file Image_2.JPEG]
